# Scalable Precision Psychiatry With an Objective Measure of Psychological Stress: Prospective Real-World Study

**DOI:** 10.2196/56086

**Published:** 2025-07-07

**Authors:** Helena Wang, Norman Farb, Bechara Saab

**Affiliations:** 1 Research Division Mobio Interactive Pte Ltd Singapore Singapore; 2 Department of Psychology University of Toronto Mississauga Toronto, ON Canada; 3 Department of Psychological Clinical Sciences University of Toronto Scarborough Ontario, ON Canada

**Keywords:** precision psychiatry, digital biomarkers, personalization, mental health, digital therapeutics, recommender system, psychological stress, therapy algorithm, psychiatry, mood, mental well-being, real world, data-driven, mental healthcare, forms, therapy, fidelity, recommendations, ecological momentary assessment, psychotherapy, sessions, self-report, patient data, users

## Abstract

**Background:**

Before meaningful progress toward precision psychiatry is possible, objective (unbiased) assessment of patient mental well-being must be validated and adopted broadly.

**Objective:**

This study aims to compare the fidelity of a precision psychiatry therapy recommendation algorithm when trained with an objective quantification of psychological stress versus subjective ecological momentary assessments (EMAs) of stress and mood.

**Methods:**

From 2786 unique individuals engaging between March 2015 and December 2022 in English language psychotherapy sessions and providing pre- and postsession self-report and facial biometric data via a mobile health platform (Mobio Interactive Pte Ltd, Singapore), analysis was conducted on 67 “super users” that completed a minimum of 28 sessions with all pre- and postsession measures. The platform used has previously demonstrated reduced psychiatric symptom severity and improved overall mental well-being. Psychotherapy recordings (“sessions”) within the platform, available asynchronously and on demand, span mindfulness, meditation, cognitive behavioral therapy, client-centered therapy, music therapy, and self-hypnosis. The platform also has the unusual ability to rapidly assess mental well-being without bias via an easy-to-use objective measure of psychological stress derived from artificial intelligence–based analysis of facial biomarkers (objective stress level [OSL]). In tandem with the objective measure, EMAs obtain self-reported values of stress (SRS) and mood (SRM). ∆OSL, ∆SRS, and ∆SRM (with delta referring to the presession subtracted from the postsession measurement) were used to independently train a therapy recommendation algorithm designed to predict what future sessions would prove most efficacious for each individual. Algorithm predictions were compared against the efficacy of the individual’s self-selected sessions.

**Results:**

The objective measure of psychological stress provided a differentiated delta for the measurement of therapeutic efficacy compared to the 2 EMA deltas, as shown by clear divergence in ∆OSL vs ∆SRS or ∆SRM (*r*<0.03), while the EMA deltas showed significant convergence (*r*=0.53, *P*<.01). The recommendation algorithm selected increasingly efficacious therapy sessions as a function of training data when trained with either ∆OSL (*F*_1,16_=5.37, *P*=.03) or ∆SRM data (*F*_1,16_=3.69, *P*<.05). However, the sequential improvement in prediction efficacy only surpassed the efficacy of self-selected therapy when the algorithm was trained using objective data (*P*<.01). Training the algorithm with EMA data showed potential trends that did not reach significance (∆SRS: *P*=.09; ∆SRM: *P*=.12). As a final insight, self-selected therapy sessions were overrepresented among the algorithmically recommended sessions, an effect most pronounced when the algorithm was trained with ∆OSL data (*F*_1,14_=30.94, *P*<.001).

**Conclusions:**

These prospective data demonstrate that a rapid, scalable, and objective measure of psychological stress, in combination with a robust recommendation algorithm, can autonomously identify clinically meaningful therapy for individuals. More broadly, this work illustrates the potential for objective data on mental well-being to improve precision psychiatry and the capacity for mental health care professionals to match global demand.

**Trial Registration:**

ClinicalTrials.gov NCT06265909; https://clinicaltrials.gov/ct2/show/NCT06265909

## Introduction

Psychiatry remains unique within medicine as the only major field that does not use objective data in standard practice.

Precision psychiatry aims to change this, pulling psychiatry into the realm of modern medicine with treatment options that match the unique profile of each patient [1]. Where used, these personalized treatment plans are predominantly informed by neuroimaging, genetic biomarkers, and medical history. However, despite great promise and considerable funding, precision psychiatry has yet to deliver major improvements for the patient [2,3]. In this study, we explore whether readily obtainable objective data on the patient’s moment-to-moment mental well-being can be used to inform a precision psychiatry therapy recommendation algorithm.

**Figure 1:**
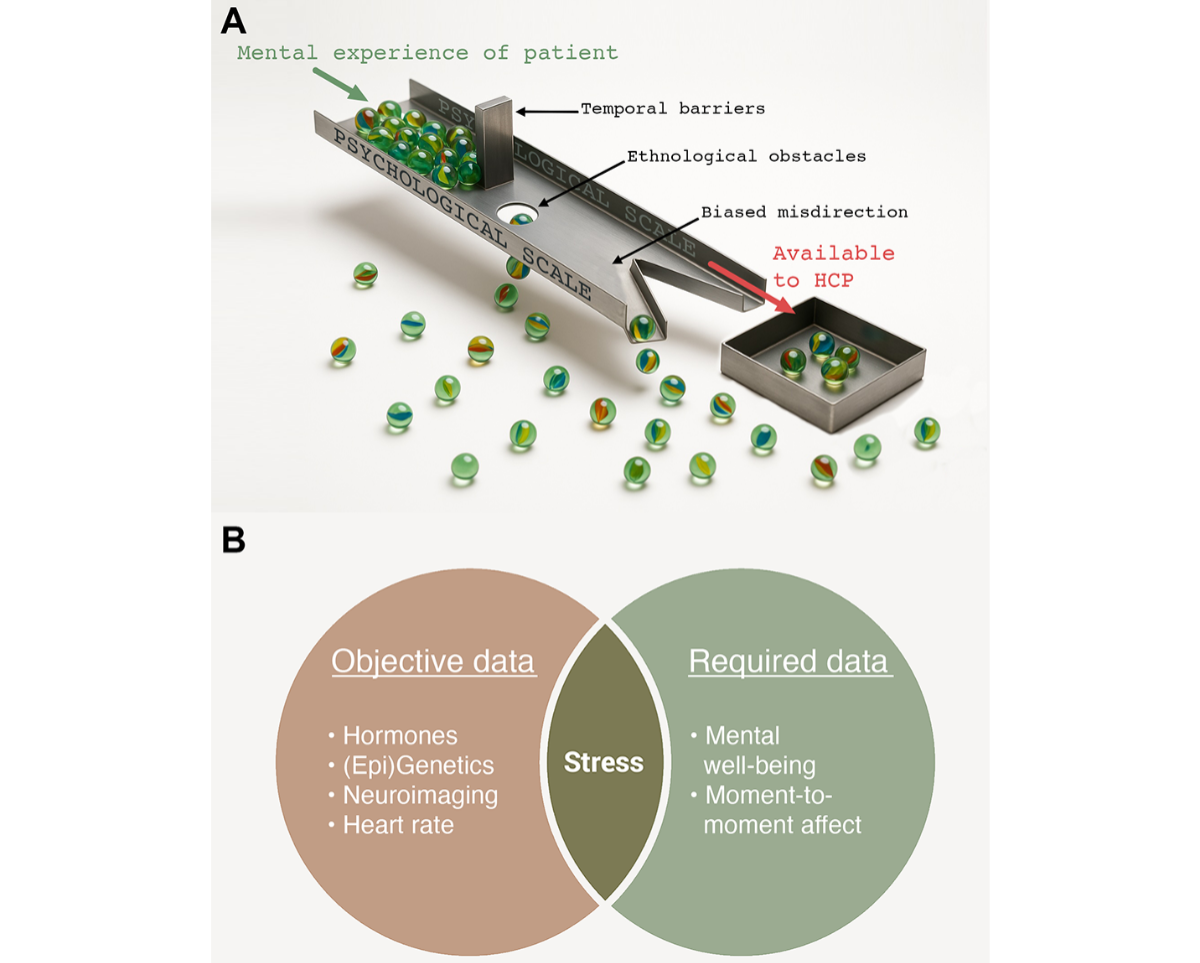
Illustrating known problems of common data sources in psychiatry. (A) Significant limitations inherent to psychological scales: (1) temporal barriers, (2) ethnological obstacles, and (3) biased misdirection. While validated scales will remain integral to the diagnosis of mental health conditions in the near term, subjective scales have less to offer precision psychiatry. (B) Venn diagram of (1) quantifiable and objective data and (2) desired and required data in precision psychiatry. Objective measures often used in precision psychiatry (left), such as neuroimaging and multiomics, have little overlap with the measures that are needed to assess treatment efficacy (right). We propose that an unbiased artificial intelligence prediction of psychological stress based on heart rate variability may represent an early data source that is both objective and required.

At present, mental well-being and psychiatric symptom severity are predominantly assessed through retrospective self-reports in the form of psychological scales. While these scales are often rigorously interrogated for face, content, and divergence reliability and validity, it is well accepted that they face profound limitations when used in the real world. These multifaceted limitations fall into 3 general classes that serve as barriers, obstacles, and misdirection, preventing health care professionals from accessing the true mental experience of their patients (Figure 1A).

First, scales have a “temporal” barrier, typically requiring about 5 to 30 minutes to complete, and are designed to retrospectively access a relatively wide window of time (eg, the previous 2 weeks). Psychological scales, therefore, cannot be administered in rapid succession or assess real-time changes in a patient’s mental well-being [4]. Second, scales impart “ethnological” obstacles since natural cultural and personality divergence means that any given psychological scale will only be maximally informative for a relatively narrow portion of the global population [5]. Third, and both most obvious and most troubling, scales can misdirect through “bias.” Subjective by design, scales contain a plethora of documented biases related to (1) recency, (2) recall, (3) expectation, (4) confirmation, and (5) response [6,7]. Response biases, in particular to gender [8] and cultural background [9,10], markedly influence the tendency to report negative affect.

Temporal barriers of psychological scales can be addressed by incorporating ecological momentary assessments (EMAs), a practice of daily or hourly self-reporting often completed by the patient via a common mobile device [11]. However, subjective by nature, EMAs do little to address the other 2 limitations of subjective measures. To address ethnological and bias limitations, objective data are essential. Already, precision psychiatry attempts to incorporate many objective measures, in particular brain imaging and (epi-)genotyping [3]. However, these data, such as medical records, are also susceptible to temporal limitations. Moreover, the objective data currently used and proposed for use in precision psychiatry [12,13], even if collected through accessible tools such as wearables, may be too far removed from the phenotype of clinical interest (mental well-being) to optimally inform precision psychiatry models (Figure 1B). We argue here that a rapid, pan-ethnological, and objective measure of mental well-being is needed to meaningfully move toward precision psychiatry that is viable at the scale required to match global demand.

Understanding the profound medical opportunity that would emerge from establishing measures of mental health that are not subject to the temporal, ethnological, and bias limitations, we developed a novel objective measure of psychological stress using deep neural network (DNN) processing of selfie video biomarkers [14]. We chose stress for 2 reasons. First, stress is a physiological process tightly linked to overall well-being [15] and directly causes or negatively impacts a broad proportion and large variety of medical conditions [16]. Second, the autonomic nervous system underlying stress influences heart rate variability (HRV), which can be directly measured at scale with remote photoplethysmography [17,18]. Indeed, due to its relationship with stress, HRV has recently gained traction in medical practice [19,20]. Our psychological stress DNN was trained with data from thousands of patients in over 150 countries, from whom heart rate and the power of HRV within 2 frequency domains (“HRV-high” and “HRV-low”) [14] were captured in real time via facial remote photoplethysmography [17]. In a previous analysis, this DNN achieved 86% accuracy and a mean squared error of 0.01, substantially outperforming the logistic regression conventionally used to estimate stress from HRV [14].

The purpose of our current study was to probe the potential utility of the DNN psychological stress data for training a precision psychiatry recommendation algorithm. The ultimate aim is to improve clinical efficacy by rapidly matching patients to their ideal therapy regimen. To this end, we made use of a commercialized mobile health platform (Mobio Interactive Pte Ltd, Singapore). The platform has been used in a variety of clinical contexts [21-33] and is one of the few software applications of its kind to outperform a placebo in a randomized controlled trial [21,30]. The platform gives patients access to over 1000 distinct audio files delivered by over 60 professionals and across 6 major languages as of January 2024. These audio files are typically 5- to 45-minute sessions that serve to support relaxation, enhance stress resilience, and otherwise stimulate neural plasticity underlying psychiatric symptom reduction. Since the mobile platform is installed on a patient’s smartphone or tablet, patients are free to engage with therapy at the time and place most convenient and effective for them. Before and after these on-demand asynchronous sessions, patients are invited to perform a 30-second selfie scan (capturing their psychological stress) and complete 2 EMAs, previously demonstrated to correlate with high significance to the principal components of relevant psychological scales [21]. The platform, therefore, provides an attractive means to investigate the fidelity of therapy recommendation algorithms.

The use of recommendation systems within mobile apps is not new; for example, see the studies by Bidargaddi et al [34], Mohr et al [35], and Cheung et al [36]. However, to our knowledge, there have been no attempts to train a recommendation algorithm with an objective measure of mental well-being. In a recent review of 73 studies on recommendation systems in health apps, approximately half (47%) did not attempt to validate the recommendations [37]. Those who did primarily used one or more self-reported measures of mood, sleep, anxiety, depression, or energy, and compared users who received personalized content recommendations to passive control groups that either received no content or received content at random. Seldom has objective data been used to train or evaluate recommendation algorithms, and in these cases, the results are thus far underwhelming. For example, tailored antismoking messages to patients with substance use disorder led to higher user ratings (subjective data) when compared with a conventional messaging system, but the tailored recommendations failed to impact smoking rates (objective data) [38].

Here, we evaluate the fidelity of a precision psychiatry recommendation algorithm when trained with objectively quantified stress, in comparison to training with 2 validated EMAs of stress and mood. In all cases, the efficacy of the recommended therapy was examined relative to the efficacy of therapy content that was self-selected by patients. This prospective study design provides the controlled methodology required to model the impact of precision psychiatry against standard care.

## Methods

### Study Design and Patient and Public Involvement

The prospective, observational real-world study accessed data from a mobile health platform (Mobio Interactive Pte Ltd, Singapore) equipped with computer vision and artificial intelligence to objectively quantify psychological stress [14,17] and benchmarked EMAs to subjectively measure stress, valence, and arousal [21]. Asynchronous and on-demand psychotherapy was available as audio files and has been clinically validated across the mental illness severity spectrum [21-24,26,27,30-33]. Users consented to the use of their anonymous data for research purposes via the platform’s Terms of Use and were not involved in a formal process to inform the design of this research. Patient identity remained anonymous throughout the study. An exception by the institutional review board for consent was granted by Advarra (Ontario, Canada). This study has been reported in line with the STROBE (Strengthening the Reporting of Observational Studies in Epidemiology) guidelines (Multimedia Appendix 1).

### Data Collection, Setting, Participants, Bias, and Study Size

Data were collected between March 2015 and December 2022 on 36,160 unique users, compliant with the HIPAA (Health Insurance Portability and Accountability Act), Personal Health Information Protection Act (PHIPA), and General Data Protection Regulation (GDPR). Of these, 2786 unique individuals engaged in biometric and self-report data collection before and after engaging with asynchronous psychotherapy. No audio or video was collected, transferred, or stored.

To protect the real-world applicability of the results, users were not given any special instructions or information about the nature or possibility of the current analyses. As a consequence, user data varied greatly in terms of engagement and app-use characteristics. To create a single, unified, and consistent dataset that could be leveraged across all intended analyses, data were filtered to include only English-language psychotherapy sessions that contained the required session payloads for algorithm inclusion (refer to the “Recommendation Algorithm” section below) and only when the objective and 2 subjective measures were completed before and after each session. A power analysis indicated a minimum of 66 unique users were required to achieve 80% power for detecting a medium effect size via the intended analyses. This limit afforded over 28 sessions with all pre- and postsession measures from 67 unique users.

### Materials and Variables

#### Objective Stress Level

Objective stress was obtained via a 30-second “selfie” video captured with the front-facing camera of a mobile device (smartphone or tablet). Computer vision data extracted from the videos in real time were passed through a DNN to compute the objective stress level (∆OSL) at that moment, as previously described in [14]. ∆OSL is represented with a value between 0 and 1, with greater values representing more stress.

#### Subjective Self-Reported Stress

Subjective stress was quantified via an animated digital “slider.” Users reported their current level of stress either by dragging a marker on the slider to a position of their choosing between “none” (0) and “extreme” (10) or by tapping on one of four faces positioned above the slider, with each face visually depicting stress levels at the midpoints of four quadrants (ie, values of 1.25, 3.75, 6.25, and 8.75). Users were instructed to input the stress that represents how they feel “right here, right now.”

#### Subjective Self-Reported Mood

Subjective mood was quantified via a “mood board,” which asked users to select from 32 different words representing various emotions (eg, “delighted,” “content,” “gloomy,” and “tense”). The mood board consists of 2 axes, one spanning from “unpleasant” to “pleasant” and the other from “mild” to “intense.” Each quadrant contains 8 mood words. Users were instructed to tap on the words that represent how they feel “right here, right now.” Unlike subjective self-reported stress (∆SRS), where users directly report a numerical value representing their stress level, subjective mood is quantified indirectly from the selected words. During data analysis, each selected word was assigned a score between –2 and +2, depending on its connotations for valence (pleasantness) and arousal (saliency). For example, the mood words “happy,” “relaxed,” “depressed,” and “nervous” were assigned scores of 2, 1, –1, and –2, respectively. An overall score of mood was then computed from the sum of the selected mood words and converted to a value between 0 and 1, where a greater score indicates better mood. Subjective self-reported mood (∆SRM) is therefore an implicit measure insofar as users are not informed how selecting words informs the overall score of their mood.

### Recommendation Algorithm

The recommendation algorithm was developed internally at Mobio Interactive in advance of conducting this study and was not altered during analysis. The algorithm uses a noncollaborative user-item interaction design, relying exclusively on pre- and postsession data from a consistent user. Prior knowledge of user characteristics (eg, gender, age, ethnicity, and medical diagnosis) is not necessary. The content selections and responses of other users were not considered and thus did not influence the algorithm’s recommendations for a given user. This algorithm design thus leverages neither collaborative filtering nor content-based filtering, instead treating each individual as an individual without prior assumptions about how therapy may influence them based on demographics, diagnoses, or the response patterns observed at large. A design of this nature was considered necessary due to the global footprint of the dataset and an understanding that how patients respond to treatment is highly idiosyncratic and unpredictable. Thus, the only assumption used by the algorithm is that the forms of therapy that are most effective for a given patient at one point in time will reliably predict the forms of therapy that will be most effective for the same patient at a later point in time.

A total of 3 types of pre- and postsession data were available to provide an evaluation of historical efficacy: ∆OSL, ∆SRS, and ∆SRM (as explained above within the “Materials and Variables” section). The algorithm was trained independently on each of these 3 measures, first by calculating the post-pre delta:

ΔWM = WMpost – WMpre **(1)**

Where WM is the well-being measure (ie, ∆OSL, ∆SRS, or ∆SRM). For measures of stress, the more negative the ∆WM, the more efficacious the session is considered for that user (and vice versa for mood). For each user, all sessions were then ranked according to efficacy.

Sessions that are intended to be directly therapeutic, that is, are not purely (psycho)educational, are each paired with a payload of (1) 3 out of a total of 36 possible “mood words” and (2) 3 out of a total of 24 possible “intent words.” The pairing of sessions to mood and intent words was conducted according to a consensus among the content creation team at Mobio Interactive and was later verified by the session’s guide (the voice within each audio recording), including clinical psychologists, psychiatrists, mindfulness teachers, and other relevant professionals involved in content creation for the platform. Mood and intent words paired to a given session were next assigned a “raw word score,” R, equal to the ∆WM value from that session.

Next, the algorithm then allowed for potentially important factors of influence to scale R:

S = R × A × B × C **(2)**

Where S is the scaled word score, and A, B, and C are scalers. At the time of analysis, one scaler was used, A, to control for the potential influence of the session guide, and according to the formula:



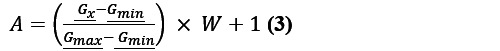



Where 
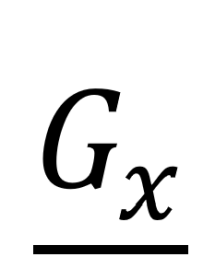
 is the mean ∆WM of the session guide for the user, 
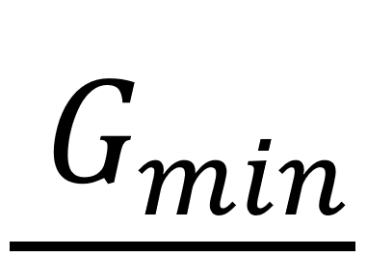
 is the mean ΔWM of sessions delivered by the guide that is least efficacious for the user, 
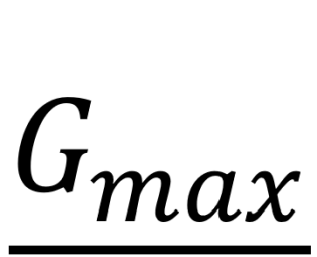
 is the mean ΔWM of sessions delivered by the guide that is the most efficacious for the user, and W is the “guide weight,” an arbitrary constant that controls the degree to which scaler A influences S. For simplicity, this analysis used W=1. Formula 3 increases the influence of mood and intent words delivered by session guides that are generally more efficacious for a given user.

Next, for each mood and intent word, S for each session was summed to produce the “total word score,” T:

T = ∑S **(4)**

Finally, the 3 mood words and 3 intent words for each user with the greatest T were input into a proprietary algorithm that ranks sessions within the entire possible library according to their association with that specific combination of 6 words. The top 3 sessions in this ranking were considered the algorithmically selected (AS) sessions for the patient.

#### Evaluation of the Algorithm

The algorithm, designed to use each user’s corpus of session data to rank the likelihood of a given session for its potential to benefit the same user, theoretically should increase its predictiveness as the user completes more sessions. To explore the effect of increasing training set size, therefore, we trained the algorithm multiple times for each user, starting with 1 session and sequentially increasing the number of training sessions by 1. At each stage, the algorithm’s predictiveness was evaluated using all remaining session data from the same user. For example, if a user completed a total of 75 sessions and the first 15 were used to train the algorithm, then sessions 16-75 were used for testing. If a user completed a total of only 35 sessions and the first 10 were used to train the algorithm, then sessions 11-35 were used for testing. Increasing the training set size thus had an inverse impact on the testing set size. The sequentially decreasing size of the testing set gave rise to statistical limitations at the tail end of this process. However, this analysis design was still preferable to alternatives since it maintained cross-comparison consistency, maximized the total size of the dataset available for analysis, and best reflected the real-world nature of the source data.

In all cases, the data type (∆OSL vs ∆SRS vs ∆SRM) used to train the algorithm was also used to evaluate the predictiveness of the algorithm. To examine if AS sessions were associated with a better outcome for the user, they were compared against “user-selected” (US) sessions using a within-subject ANOVA. The choice to include all US sessions (including those that could have been simultaneously selected by the algorithm) was made to ensure the highest bar for comparison. The comparison design may underestimate the difference between AS and US sessions, but it best reflects the real-world nature of the source data and the real-world application of these types of health care solutions. Comparison against self-selection was, thus, in our view, the only meaningful choice. To compare results across various well-being measures, data were *z* score normalized.

#### Cross-Validation

ANOVA comparisons were 10-fold cross-validated using a bootstrapping approach by randomly sampling 80% (n=54) of the users and repeating the same training and testing on this reduced sample.

### Statistical Analysis Software

All statistical analyses were conducted using R (version 4.0.2, R Core Team).

### Ethical Considerations

The study protocol was reviewed and approved by an independent review board (Pro00084303; Advarra; Ontario, Canada). Participant safety and privacy were protected through standard operating procedures defined in the DCB0129 and ISO27001 certifications for software as medical software and cybersecurity, respectively, awarded to the developer (Mobio Interactive, Singapore).

## Results

### Participants and Descriptive Data

Of the 67 patients eligible for analysis, 43 (64%) identified as female, 19 (28%) identified as male, and 5 (7%) selected “other” under gender. Self-reported age ranged from 18 to 66 years, with the largest age group being 25-34 years (n=21, 31%), and the smallest being 18-24 years (n=7, 10%). Geolocation data indicated predominant use in Australia (n=27, 40%), New Zealand (n=12, 18%), and Canada (n=11, 16%). The mean number of sessions completed by each user (with or without pre- and postsession measures) was 154.9 (SD 228.1, median 76, range 40-1328). English-language sessions with all pre- and post-session measures completed by the participants had a mean of 42.7 (SD 16.8, median 37, range 28-108).

### Asynchronous on-Demand Therapy Is Beneficial for Patients

Inspection of the 3 measures intended to train the recommendation algorithm suggested a mean reduction in stress and an improvement in mood across all sessions. In all cases, the CIs did not include zero, as shown by ∆OSL –0.003 (95% CI –0.026 to –0.00091), ∆SRS –0.09 (95% CI –0.12 to –0.0075), and ∆SRM 0.13 (95% CI 0.01-0.24). These real-world data corroborate findings from controlled clinical investigations [21-24,26,27,30-32].

### Divergence Between Objective and Subjective Data

**Figure 2:**
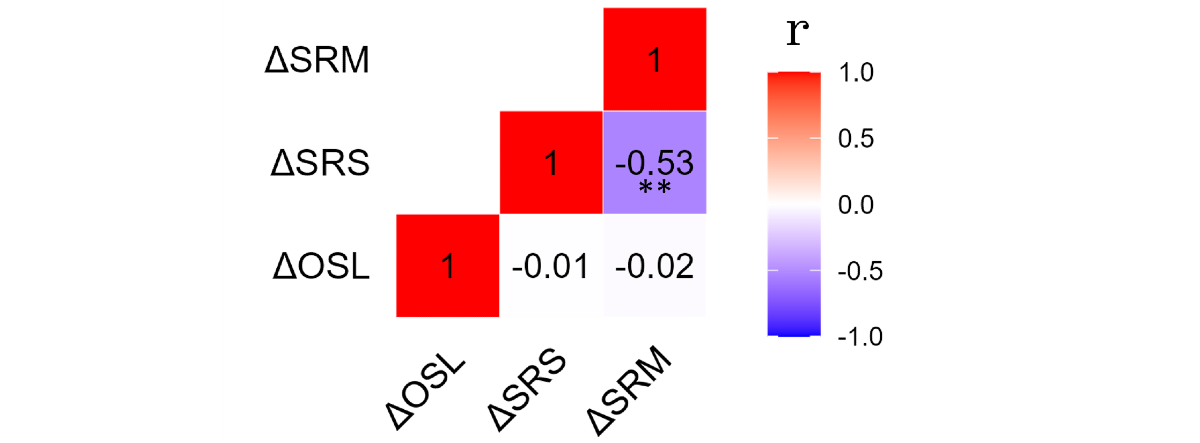
Correlation analysis between the 3 wellness measure deltas used in the prospective study. Patients were monitored on a fully voluntary basis for years, without specific direction on how often they should complete assessments. Assessments were only included in the analysis if all 3 were completed in tandem. Pearson correlation analyses revealed a significant negative correlation between the deltas of the 2 ecological momentary assessments (EMAs), indicating that psychotherapy-associated decreases in self-reported stress (∆SRS) are accompanied by a corresponding increase in self-reported mood (∆SRM), as calculated by sentiment analysis of the patient’s “mood word” selections. In contrast, the delta of objective stress level (∆OSL), which exclusively relies on facial biomarkers obtained through a 30-second selfie scan, did not correlate with either of the 2 EMA deltas. Thus, while the objective stress measure has been previously reported to closely track ∆SRS, the change that results from engaging in psychotherapy represents a distinct data source that may be highly valuable for precision psychiatry applications. Values indicate Pearson correlation, r; *P*<.01. OSL: objective stress level; SRM: self-reported mood; SRS: self-reported stress.

Pearson correlations between the 3 measures intended to train the recommendation algorithm suggested independence between the objective measure and the 2 subjective measure deltas. ∆SRS and ∆SRM correlated significantly with each other, but neither correlated with ∆OSL (Figure 2). The objective measure, at least within this mental health platform, may therefore be a valuable, differentiated source of information about how mental well-being is impacted by therapy in real time.

### Objective Data Were Superior to Subjective Data for Therapy Recommendation Algorithm Training

Algorithm performance was evaluated using linear regression analysis, within-subject ANOVA, and 10-fold bootstrapping to compare the efficacy of AS sessions against US sessions, using the same well-being measure (∆OSL, ∆SRS, or ∆SRM) for both training and testing.

**Figure 3:**
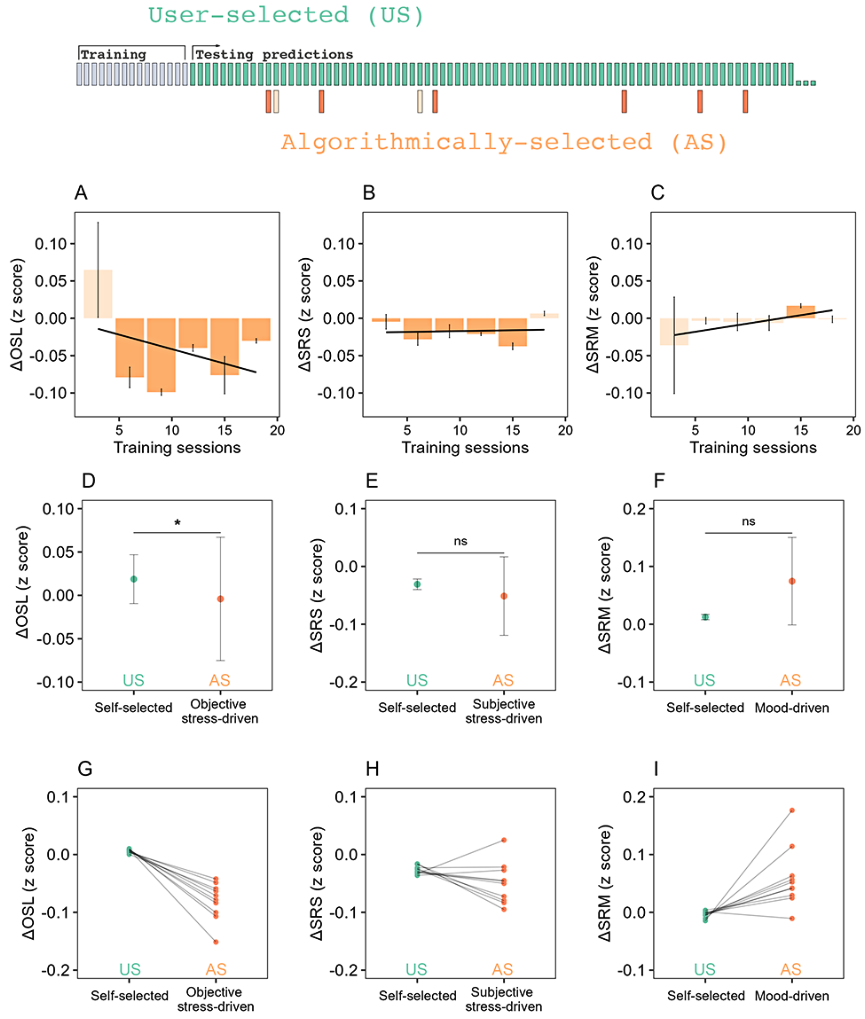
Performance analysis of a precision psychiatry recommendation algorithm in a prospective real-world study when the algorithm was trained with 3 separate measures of well-being. Patients were monitored for years using a mobile health platform, on a fully volunteer basis, and without specific direction as to how often they should complete assessments. Similarly, no specific guidance was provided on what psychotherapy sessions might be best for each patient, providing the patients instead with free choice to decide when and how they engaged with the platform. (A)-(C): The differential of efficacy when completing AS and US psychotherapy sessions as a function of the training set size. For stress measures (A and B), values below zero (dark orange) indicate that the AS sessions are more efficacious than the US sessions. For the measure of mood (C), values above zero (dark orange) indicate that AS sessions are more efficacious than US sessions. (D)-(F): Comparisons of the efficacy of AS and US sessions, with 15 sessions included in the training set. When the precision psychiatry algorithm was trained with objective stress data, the AS psychotherapy sessions proved to be more efficacious (were accompanied by larger decreases in stress) than the US psychotherapy sessions. These results demonstrate that objective real-time measures of psychological stress can be used to improve psychotherapy treatment recommendations in a highly scalable and autonomous manner. (G)-(I): Cross-validation of (D)-(F) via 10-fold bootstrapping, taking 80% of users for each sample, demonstrated an absence of oversampling. Error bars represent the SE of the mean. *P<.05. AS: algorithmically selected; OSL: objective stress level; SRM: self-reported mood; SRS: self-reported stress; US: user-selected.

When ∆OSL or ∆SRM data were used, AS sessions demonstrated increasing efficacy relative to US sessions as a function of training set size, as revealed by linear regression analysis (∆OSL: *F*_1,16_=5.37, *P*=.03; ΔSRM: *F*_1,16_=3.69, *P*=.50; Figures 3A and 3C). No effect of training set size was observed when ∆SRS data were used (*F*_1,16_=0.046, *P*=.83, Figure 3B).

Within-subject ANOVA comparisons allowing the algorithm to “learn” from 15 training sessions revealed that ∆OSL training data informed an algorithm that recommended significantly more efficacious sessions when compared with US sessions (*F*_1,66_=8.22, *P=*.005; Cohen *d*=0.17; Figure 3D). In contrast, the same comparisons when self-reported data were used did not reach significance (∆SRS: *F*_1,66_=1.24, *P=*.09, Cohen *d*=0.095; ∆SRM: *F*_1,66_=2.21, *P=*.12, Cohen *d*=0.041; Figures 3E and 3F).

Ten-fold bootstrapping, consistent with the ANOVA results, suggested an absence of oversampling (Figures 3G-I).

### Patients Gravitate Toward More Efficacious Sessions

**Figure 4:**
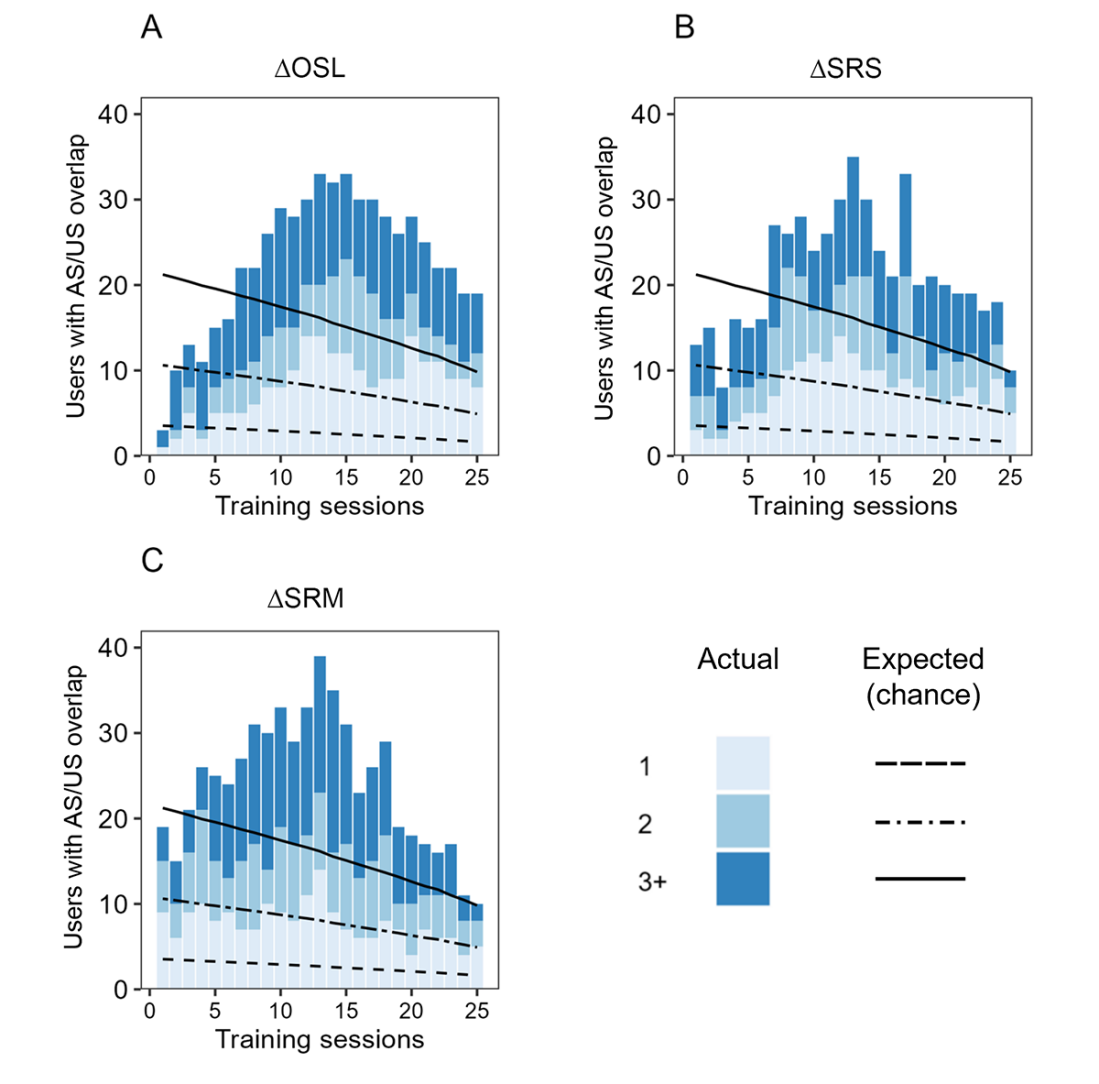
Overlap of algorithmically selected (AS) and user-selected (US) sessions in the testing data when the recommendation algorithm was trained with (A) objective stress (∆OSL), (B) self-reported stress (∆SRS), and (C) self-reported mood (∆SRM). Patients were granted free selection of psychotherapy content from a mobile health platform, and the precision psychiatry recommendation algorithm was designed to iteratively predict which 3 sessions from the entire available library would be most efficacious for each patient. The expected (by chance) proportion of AS sessions being present in the US sessions is plotted using straight lines, with each line corresponding to the chance of observing (1) one, (2) two, or (3) three or more AS sessions in the testing dataset, and as the training dataset increased from 1 to 25 sessions. The actual proportion of AS sessions is shown using the underlying bar plot. In all three cases, and most pronounced when the algorithm was trained with objective data, more overlap was observed than what would be expected by chance. This indicates that patients tend to gravitate toward sessions that are objectively more efficacious, even if they were not necessarily aware of doing so. AS: algorithmically selected; OSL: objective stress level; SRM: self-reported mood; SRS: self-reported stress; US: user-selected.

The purpose of the recommendation system explored in this study is to ensure that therapy sessions delivered to patients are as efficacious as possible. This being the case, we were curious to examine the overlap of AS and US sessions. Post hoc analysis via an ANOVA using the expected versus actual quantity of sessions with overlap (1, 2, and 3+) revealed a sharp rise in the number of patients with overlap between AS and US (Figure 4). This rise in overlap quickly exceeded chance (∆OSL: *F*_1,14_=30.94, *P*<.001; ΔSRS: *F*_1,14_=19.33, *P*<.001; ΔSRM: *F*_1,14_=67.17, *P*<.001) and followed a quadratic relationship as revealed by regression analysis (∆OSL: *F*_2,22_=204.1, *P*<.001; ΔSRS: *F*_2,22_=23.98, *P*<.001; ΔSRM: *F*_2,22_=51.55, *P*<.001). These results may hint that users intuitively know, perhaps subconsciously, which sessions are providing benefit.

## Discussion

### Principal Findings

In this study, we trained and evaluated a recommendation algorithm with an objective measure of psychological stress. We also performed the identical analyses using 2 EMAs. We compared the efficacy of algorithmically recommended sessions against sessions selected by patients themselves. Our results indicate that a precision psychiatry recommendation algorithm, when trained with an objective measure of psychological stress, can faithfully predict what forms of therapy will be more efficacious on average than the therapy patients will choose for themselves. The same clinical benefit of recommendations was not observed when the algorithm was trained with subjective EMA data.

To our knowledge, this is the first prospective study that leverages a rapid and scalable objective measure of well-being to train and evaluate a precision psychiatry therapy recommendation algorithm. The results demonstrate the predictive ability of the approach and provide a framework for statistical analysis, measurement methodology, and algorithm design in the pursuit of precision psychiatry at a scale that meets global demand. Several interesting and, at times, surprising observations emerged during our analysis.

First, patients garnered a mean benefit from engaging with content on the platform. This observation is an important real-world corroboration of previous research [21-24,26,27,30-33]. Without a strong foundation of efficacy from mobile delivery of asynchronous and on-demand therapy, recommendation algorithms embedded within these products are of no value to patients or health systems.

Second, the deltas in subjective versus objective data appear to measure separate therapy-response phenomena (Figure 2). Additional support for divergence between objective and subjective data deltas was also revealed in finding that users consistently self-reported larger changes to their well-being than what was revealed by objective data. Similar divergence between objective and subjective measures of mental well-being has been reported by others [39] and may arise in part from the susceptibility of self-reported data to biases of recall, confirmation, response, and expectation. While EMAs may circumvent recall bias, the biases of confirmation, expectation, and response likely persist. In addition, EMAs rely on a precise awareness of one’s immediate psychological states, which likely varies between (and within) individuals. Meanwhile, convergence of the EMAs suggests the potential for diminishing returns when collecting multiple sources of self-reported data, at least when compared with a divergent measure like the psychological stress DNN. Thus, irrespective of the mechanism underlying divergence between objective and subjective well-being data deltas, their dissociation may serve as an independent rationale for including objective data on mental well-being in the practice of psychiatry.

Third, objective data on psychological stress captured immediately before and after therapy sessions were sufficient to train an algorithm to prospectively predict what therapy sessions would be more efficacious for a patient compared with the content patients would choose for themselves (Figures 3A, 3D, and 3G). This finding is a realization of the general potential for objective measures of well-being in psychiatry. Beyond facial biomarkers, other methods, such as biomarker analysis of speech [40], will likely be critical. Being less susceptible to biases and independent from interoception accuracy, objective data could be more sensitive to factors important for predicting well-being outcomes [41,42]. The finding that an objective measure of stress can train an algorithm to select ideal content for patients is especially exciting considering its rapidity of collection (30 s) and potential for broad adoption within health care since the software runs on hardware most patients have with them all day (smartphones).

Fourth, subjective data in this study were not sufficient to train the recommendation algorithm to an extent that its predictions were statistically superior to self-selected sessions (Figures 3E and 3F). While other studies have reported success with recommendation algorithms trained with subjective data [34], these studies generally compared algorithm recommendations against randomly selected content (instead of the more meaningful patient-selected content, as was the case in our analysis). As discussed in the “Introduction” section, the limitations of subjective data for algorithm training are likely rooted in the multifarious, well-described biases. In particular, an expectation bias was easily observable in our dataset (Figure 3D-3F, green markers) and may have impaired the utility of self-report data for training the recommendation algorithm. It is also possible that subjective data have greater session-to-session variability (more noise), giving rise to a less consistent training dataset.

Fifth, in self-reports, when the recommendation algorithm was trained with ∆SRM data, algorithm predictiveness improved as a function of the number of sessions in the training dataset (Figure 3C). No such relationship was found for ∆SRS (Figure 3B). This result may be due to the direct versus implicit nature of the stress slider versus the mood board EMAs, respectively. The conclusion, if these results can be generalized, is that the less subjectivity inherent to a given measure, the better it may be for recommendation algorithm training.

Sixth, there was a surprising overlap of AS and US sessions beyond what would be expected by chance. This finding may suggest that patients intuitively gravitate toward more efficacious therapy regimens. While this is surely a comforting finding for both health care professionals and patients, we have little insight into why this overlap occurs. We did note that patients completed identical sessions multiple times, potentially signaling a preference to repeat sessions that led to greater feelings of well-being. Even if patients were not consciously aware of the subtle response differences to various forms of content, they nevertheless appear to act as their own recommendation algorithm. More simply put, the sessions patients “like” tend to also be more beneficial. The power of an accurate automated recommendation algorithm lies in improving upon this process regardless of an individual’s ability to self-select efficacious content or gain access to a clinical expert, thereby rendering the delivery of appropriate care more equitable across patient populations.

The work here demonstrates an opportunity for objective measures of well-being, such as psychological stress when rapidly and accurately obtained via a smartphone camera [14], to meaningfully inform precision psychiatry therapy recommendation algorithms. There are additional, broader benefits for psychiatric practice. In particular, removing bias will facilitate higher accuracy and standardization, ensuring a more uniform approach to diagnosis and treatment. Similarly, with the ability to observe measurable impact in real time, patients may become more engaged in beneficial treatment regimens. Objective measures may also lead to earlier detection, allowing intervention before issues become so severe they are undeniable or result in serious harm.

More broadly, using objective well-being data may reduce the stigma associated with an “invisible” self-reported condition and place mental health on the same level as physical health. When readily accessible for clinicians, objective data obtained remotely before a consultation may also reduce the time clinicians spend collecting patient data in person, freeing up time for clinicians to better understand and connect with their patients on a personal level. Objective data will also facilitate the proper inclusion of mental health into the broader health care economy, given that the payors of health care services are reluctant to cover costs that lack standardization and are susceptible to “gaming.” Finally, as we show here, objective data will facilitate better personalization of mental health care as recommendation methods continue to improve.

Our future work within precision psychiatry may explore the impact of additional weighted scalers for the current recommendation algorithm, as well as other algorithm designs, such as indication-specific content filtering. For example, patients with major depressive disorder may respond best to consistency with the therapy guide, while patients with generalized anxiety disorder may be particularly influenced by the degree of silence afforded to them during a given therapy session. Thus, there is room to introduce additional content classifiers for consideration by the algorithm. Separately, the efficacy of psychotherapy may be moderated by various demographic factors, including cultural background [43]. Mindfulness training and meditation courses are indeed often adapted for specific clinical populations [44-46], age groups [47,48], and cultural groups [49-51]. Incorporating diagnoses, medical history, and demographic factors into the algorithm design may lead to further improvement in its predictions. However, these types of data can also introduce undesired biases into the algorithm that counteract our intent to balance functionality across individuals. Caution will therefore be required, keeping in mind that content filters may not impart any net benefit.

Future analysis may also examine additional objective measures of well-being beyond psychological stress. In particular, biomarkers from microexpressions and facial blood distribution could be leveraged to produce accurate and objective measures of affect across the domains of valence and arousal. Once validated, multiple objective measures may prove synergistic in their ability to capture the psychological profile of patients as they engage with therapy and tailor treatment recommendations with ever-increasing accuracy.

### Limitations

There are a few limitations to this study. First, while our DNN quantifies stress from HRV with unprecedented precision [14], the use of facial biomarkers as the ultimate data source requires patients to actively engage with each measurement by way of a mobile selfie scan, ultimately limiting the length of the analysis period and complicating data capture during therapy (as opposed to before and after). Additional technology developments are underway to facilitate continuous passive facial biomarker data capture without direct and continuous engagement from the patient. Moreover, the increased convenience and accessibility incurred from avoiding wearables, along with the additional data that can be captured from the human face compared with the finger or wrist, make the facial biomarker method preferable for precision psychiatry applications at a global level. Second, the dataset used in the current study was restricted to English therapy sessions, and therefore, most of these patients likely use English as a first language. This constraint was necessary to ensure homogeneity among the therapeutic content. While there are no immediately obvious reasons why the results with English content would not generalize to other languages, the restriction does create a bias to a certain patient demographic. It would be prudent and worthwhile to replicate the analyses with non-English therapy sessions once the prerequisite volume of data is at hand.

While our analysis focused on the clinical utility of leveraging objective versus subjective data on patient well-being for applications within precision psychiatry, the study design and clinical implementation of the technology also demand that the recommendation algorithm training data be obtained rapidly and at scale. Rapidity is essential since the likelihood of patients completing assessments is reduced as a direct function of how long the assessment takes. Meanwhile, scalability is essential to maximize the breadth of patients that can meaningfully benefit from the technology. Wearables and other medical hardware are major barriers for underserved populations, especially in lower socioeconomic regions of developed nations and throughout low- and middle-income countries and lower-income countries. The general theme is removing barriers and increasing convenience.

### Conclusions

Globally, more than 10% of people today need some form of mental health care, and more than 50% of the global population will require mental health care at some point in their lives [52]. This study demonstrates that effective and accessible precision psychiatry can be delivered at scale. We hope our work helps prepare the field to join the rest of medicine in consulting objective data as a regular course of action in the pursuit of optimal patient care. We also hope efficiencies generated via the technology will allow health care professionals to spend more time with their patients.

In summary, we present evidence that artificial intelligence–derived objective stress data, when captured through a mobile device’s front-facing camera before and after asynchronous therapy sessions, can identify the forms of therapy that are most efficacious for each patient and accurately predict which future therapy sessions will result in the most clinical benefit for each patient. While we feel this finding marks a major step toward precision psychiatry at scale, it is imperative to keep in mind that precision psychiatry recommendation algorithms are, in fact, recommendations. No in silico solution, no matter how accurate and powerful, will be able to adequately address all the needs of a patient.

## Appendix

Multimedia Appendix 1 STROBE checklist.

## Abbreviations

AS: algorithmically selected
DNN: deep neural network
EMA: ecological momentary assessment
GDPR: General Data Protection Regulation
HIPAA: Health Insurance Portability and Accountability Act
HRV: heart rate variability
OSL: objective stress level
PHIPA: Personal Health Information Protection Act
SRM: self-reported mood
SRS: self-reported stress
STROBE: Strengthening the Reporting of Observational Studies in Epidemiology
US: user-selected
WM: well-being measure

## Data Availability

The datasets generated or analyzed during this study are not publicly available due to restrictions outlined in the platform’s Terms of Use and Privacy Policy but are available from the corresponding author on reasonable request.
